# Clinical Outcomes of Early Administration of Proprotein Convertase Subtilisin/Kexin Type 9 Inhibitors in East Asian Patients with Acute Ischemic Stroke: A Systematic Review and Meta-Analysis

**DOI:** 10.3390/jcm15135169

**Published:** 2026-07-02

**Authors:** Sarah Alqhtani, Hannah Abid, Montaha Almatrafi, Amal Bamehriz, Shatha Alqurashi, Ahmed Alkhiri, Norah Alqhtani, Gadi Sindi, Kamal Bin Salama, Faris Alzahrani, Adel Alhazzani

**Affiliations:** 1 College of Medicine, King Saud Bin Abdulaziz University for Health Sciences, Jeddah 21423, Saudi Arabia; 2 Ministry of National Guard–Health Affairs, Jeddah 11426, Saudi Arabia; 3 Neuroscience Center of Excellence, King Faisal Specialist Hospital and Research Center, Riyadh 11211, Saudi Arabia; 4 College of Medicine, Ibn Sina National College for Medical Studies, Jeddah 22421, Saudi Arabia

**Keywords:** PCSK9 inhibitor, lipid lowering, acute ischemic stroke, early neurological deterioration, stroke recurrence, all-cause mortality

## Abstract

**Background:** Dyslipidemia is a modifiable risk factor and predictive biomarker for acute ischemic stroke (AIS) that necessitates early, aggressive lipid-lowering therapy to achieve target low-density lipoprotein cholesterol (LDL-C) levels for primary and secondary prevention. In certain patients, this can be difficult to achieve with statins alone. Proprotein convertase subtilisin/kexin type 9 inhibitor (PCSK9i) lipid-lowering agents may improve outcomes when introduced early. This review assessed whether early PCSK9i administration (within 3 weeks of AIS) reduced early neurological deterioration (END), recurrent stroke/transient ischemic attack (TIA), poor functional outcomes, and mortality. **Methods:** This systematic review and meta-analysis included randomized clinical trials (RCTs) and observational studies. Random-effects meta-analysis and subgroup and sensitivity analyses were used to assess whether effects differed by treatment timing (≤72 vs. >72 h) and study design. **Results:** Eight studies (three randomized clinical trials) in East Asian cohorts were included. Early PCSK9i initiation significantly reduced END compared with usual care (odds ratio [OR]: 0.39; 95% confidence interval [CI]: 0.26–0.57). Stroke/TIA recurrence and all-cause mortality within 6 months of stroke were also significantly reduced in the PCSK9i group (OR: 0.47; 95% CI: 0.28–0.77 and OR: 0.33; 95% CI: 0.15–0.72, respectively), and early initiation was associated with a greater likelihood of good functional outcomes at 90 days (OR: 2.28; 95% CI: 1.48–3.51). Sensitivity analyses yielded consistent results. **Conclusions:** Early PCSK9i initiation within 3 weeks of AIS onset was associated with lower rates of END, recurrent stroke/TIA, and mortality, although the certainty of evidence was limited by the small number of included studies and the predominantly observational data. Outcomes did not differ significantly by initiation timing within this period. Large-scale trials in diverse populations are needed to define the optimal initiation window and long-term clinical effects.

## 1. Introduction

Acute ischemic stroke (AIS) is defined as a sudden onset of neurological deficits resulting from focal cerebral, spinal, or retinal infarction [1]. Dyslipidemia, particularly elevated low-density lipoprotein cholesterol (LDL-C), is a major risk factor for AIS and is commonly used as a predictive biomarker for stroke risk [2]. Consequently, clinical guidelines endorse a “lower-is-better” strategy for LDL-C levels for primary and secondary stroke prevention [3]. This is typically achieved through early initiation of treatment with statins [4]; however, attaining target LDL-C levels can be difficult in some patients, as the maximum required dose to achieve optimal reduction may be intolerable due to adverse effects [3]. In such cases, combining non-statin lipid-lowering agents, such as proprotein convertase subtilisin/kexin type 9 inhibitors (PCSK9is), with statins can produce synergistic effects and mitigate statin-related adverse effects [5]. Moreover, early addition of PCSK9i to statin therapy reduces early neurological deterioration (END) [6,7] and stroke recurrence [8,9] and improves functional outcomes following AIS [10].

Existing studies have largely addressed PCSK9i use in chronic atherosclerotic disease or acute coronary syndrome (ACS); however, no comprehensive review has examined the effects of early PCSK9i initiation in AIS, in which the ischemic penumbra is most vulnerable to early deterioration. Therefore, in this systematic review and meta-analysis, we aimed to evaluate the effect of early PCSK9i administration (within 3 weeks of AIS onset) on END, recurrent stroke and/or transient ischemic attack (TIA), functional outcomes, and mortality in AIS.

## 2. Methods

This study adhered to the Preferred Reporting Items for Systematic Reviews and Meta-Analyses (PRISMA) guidelines. The PRISMA checklist is presented in Supplementary Tables S3 and S4. The study was conducted according to a pre-specified protocol registered in the International Prospective Register of Systematic Reviews (Registration: https://www.crd.york.ac.uk/prospero/ (accessed on 24 February 2026); ID: CRD420251000814).

### 2.1. Search Strategy and Selection Criteria

Databases (Medline/PubMed, Web of Science, Ovid-Embase, Cochrane Library, Scopus, Google Scholar, and the Virtual Health Library) were systematically searched from their inception to 14 March 2025. The search strategy (Table S1) combined keywords and Medical Subject Headings terms related to ischemic stroke and PCSK9is, including alirocumab and evolocumab. Prior reviews and bibliographies were manually screened to identify additional eligible articles. No restrictions on language or publication date were applied to maximize the inclusion of all relevant data. Only peer-reviewed articles were included. Two non-English studies were translated into English by the medical library at our institution to minimize language bias.

Clinical trials and observational studies were included if they met the following criteria: (1) patients aged ≥18 years diagnosed with AIS of atherosclerotic or cardioembolic origin; (2) early administration of PCSK9is (within 3 weeks of AIS onset); (3) presence or absence of a control group receiving placebo or standard of care (SOC) treatment; and (4) evaluation of END, recurrent stroke and/or TIA, functional outcomes, mortality, or changes in lipid profile following intervention. The exclusion criteria were as follows: (1) case reports, case series, animal studies, or literature reviews; (2) unclear timing of stroke onset or PCSK9i administration or if treatment was initiated beyond 3 weeks; and (3) conference abstracts or ongoing trials without a published full-text article.

### 2.2. Study Selection and Data Extraction

After removing duplicate records, titles and abstracts were independently manually screened by two reviewers selected from the author group (H.A., M.A., A.B., Sh.A., and N.A.) to identify eligible articles. Any discrepancies were resolved by consultation with a third reviewer. The same procedure was applied during full-text screening. Two reviewers then independently manually extracted data from the included studies, and any disagreements were resolved through discussion. Extracted data included study characteristics, patient demographics, intervention details and associated adverse events (AEs), and changes in clinical and laboratory outcomes following treatment.

### 2.3. Study Outcomes

Primary outcomes included all-cause mortality, stroke/TIA recurrence within 6 months, and incidence of END (a ≥2-point increase on the National Institutes of Health Stroke Scale [NIHSS] or 1-point increase in motor function within 7 days of AIS onset). Secondary outcomes included changes in 90-day modified Rankin scale (mRS) scores, with a good functional outcome defined as mRS 0–2 at 90 days, LDL-C levels from baseline, and AEs.

### 2.4. Risk-of-Bias and Quality Assessment

The quality of the included randomized controlled trials (RCTs) was assessed using the revised Cochrane Risk of Bias tool for randomized trials. Ratings in its five domains were used to classify each trial as low risk, some concerns, or high risk [11]. The Newcastle–Ottawa scale was used to evaluate the quality of observational studies. Each study was assigned a score ranging from 0 to 9 based on selection, comparability, and outcome/exposure domains. A total score ≥7 indicated good quality; 5–6, fair quality; and <5, poor quality [12]. Two authors independently evaluated the risk of bias for each study, and a third author was consulted to resolve any disagreements.

### 2.5. Statistical Analysis

Statistical analyses were conducted using Review Manager (RevMan) version 9.4.2 [13]. Most studies reported outcomes as means and standard deviations. For studies presenting LDL-C levels as medians and interquartile ranges, corresponding means and standard deviations were estimated using the method proposed by Wan et al. [14]. In addition, LDL-C values reported in mg/dL were converted to mmol/L (÷38.67). Continuous variables were expressed as mean differences (MDs), whereas dichotomous outcomes were analyzed as odds ratios (ORs) with 95% confidence intervals (CIs) based on event counts or total participants in each group. Random-effects meta-analysis with inverse-variance weighting was utilized for all outcomes to account for potential heterogeneity.

Heterogeneity was assessed using the I^2^ statistic (proportion of total variability between studies due to heterogeneity) and the Cochran Q test. An I^2^ ≥ 50% or *p* < 0.05 was considered to indicate significant heterogeneity. Because of the limited number of studies included in each analysis, publication bias was not assessed. Subgroup and sensitivity analyses were conducted to investigate the effects of treatment timing (≤72 vs. >72 h) on all-cause mortality and recurrent stroke/TIA and study design (RCT vs. observational study) on END, all-cause mortality, and recurrent stroke/TIA. Additionally, a subgroup analysis excluding studies with converted LDL-C levels was performed.

## 3. Results

### 3.1. Study Selection

**Figure 1:**
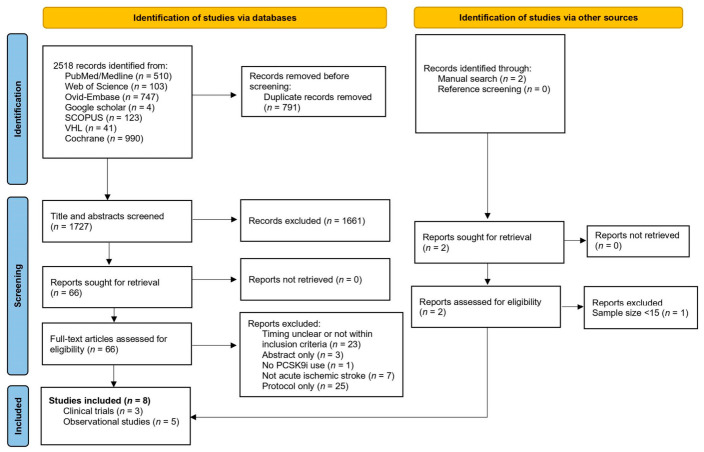
Preferred Reporting Items for Systematic Reviews and Meta-Analyses flow diagram of study selection. VHL, Virtual Health Library; PCSK9i, proprotein convertase subtilisin/kexin type 9 inhibitor.

Overall, 2518 records were identified; 1 additional study was obtained through manual screening. After removing 791 duplicates, 1727 records were screened by title and abstract. Sixty-six articles underwent full-text review; 59 were excluded because of the following reasons: unclear or delayed medication timing beyond 3 weeks after stroke onset (*n* = 23), abstract only (*n* = 3), non-acute AIS populations (*n* = 7), absence of PCSK9i use (*n* = 1), and study protocol only (*n* = 25). Eight studies met the inclusion criteria and were included in the final synthesis (Figure 1).

### 3.2. Study Characteristics

Table 1 summarizes the characteristics of the eight included studies. Among these, four were retrospective studies, three were RCTs, and one was a prospective study. The number of patients per study varied significantly (120–661). The proportions of male patients were 22.4–72%, with mean/median ages ranging from 60 to 69 years. Most studies (*n* = 6) primarily involved patients with large artery atherosclerosis and those within 24–72 h of stroke onset.

### 3.3. Patients’ Baseline Characteristics

Among the vascular risk factors, hypertension yielded the highest prevalence (60.8–83.3%), followed by diabetes mellitus (18.7–45.8%), coronary artery disease (6.67–41.6%), and previous ischemic stroke (9.0–37.6%). The mean or median baseline LDL-C levels ranged from 2.46 to 3.55 mmol/L (Table 2).

### 3.4. Intervention Details

All included studies investigated PCSK9i as an add-on therapy to the standard lipid-lowering agents: statins and/or ezetimibe. Evolocumab was typically administered at 240 mg monthly or 140 mg every 2 weeks; alirocumab was given either as a single dose or at 75 mg every 2 weeks. Most control groups received high-intensity statin treatment with or without ezetimibe. Timing of treatment initiation varied from 24 h to 14 days following AIS onset. Outcome measures primarily assessed lipid profiles, stroke/TIA recurrence, END, and functional outcomes (90-day mRS score). These outcomes were reported at different timepoints (Table 3).

### 3.5. Quality Assessment

**Figure 2:**
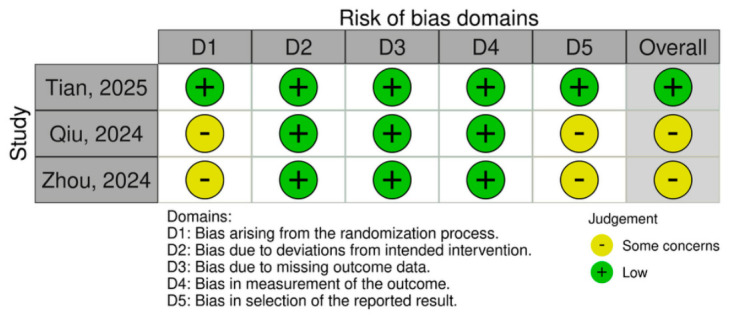
Risk-of-bias summary of included randomized controlled trials assessed using the Cochrane Risk of Bias 2 tool. “Overall” indicates the overall risk-of-bias judgment for each included study [7,15,16].

**Figure 3:**
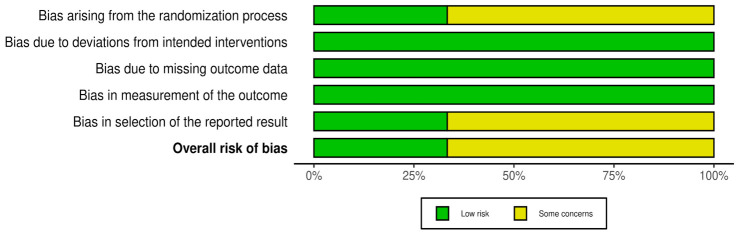
Risk of bias of randomized controlled trials assessed using the Cochrane Risk of Bias 2 tool.

Only one RCT was rated as having a low risk of bias [7], whereas the other two showed some concern due to randomization processes and potential selective reporting [15,16]. All five observational studies were deemed to be of good quality (Figure 2 and Figure 3, Table 4).

### 3.6. Primary Outcomes: Incidence of END, All-Cause Mortality, and Recurrence of Stroke or TIA

**Figure 4:**
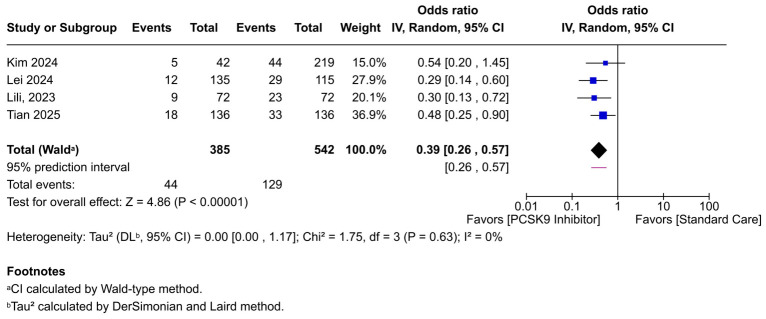
Forest plot of early neurological deterioration within 7 days. Random-effects meta-analysis comparing administration of PCSK9is versus usual care for the prevention of early neurological deterioration within 7 days after acute ischemic stroke. PCSK9i, proprotein convertase subtilisin/kexin type 9 inhibitor; CI, confidence interval [6,7,10,18].

**Figure 5:**
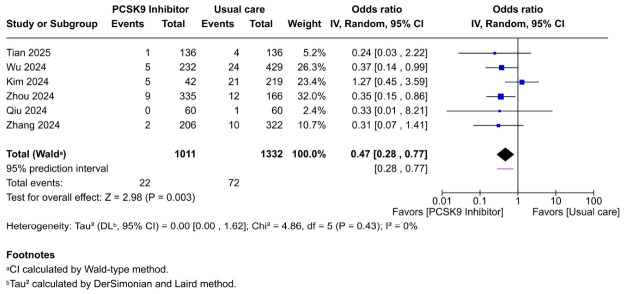
Forest plot of recurrent stroke or transient ischemic attack within 6 months. Random-effects meta-analysis evaluating the effect of early PCSK9i initiation on the recurrence of stroke or transient ischemic attack within 6 months following acute ischemic stroke. PCSK9i, proprotein convertase subtilisin/kexin type 9 inhibitor; CI, confidence interval [7,9,10,15,16,17].

**Figure 6:**
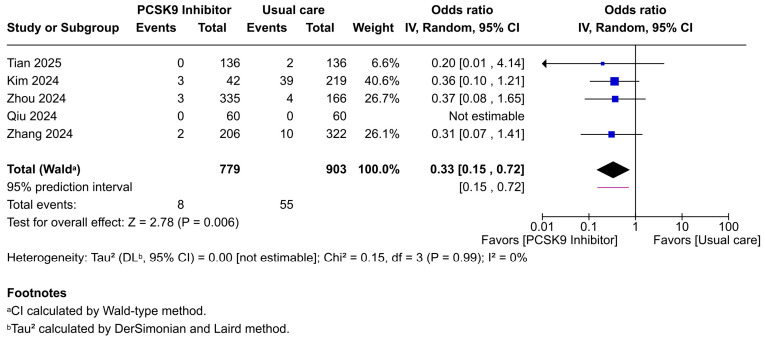
Forest plot of all-cause mortality within 6 months. Random-effects meta-analysis comparing early PCSK9i therapy with usual care for the outcome of all-cause mortality within 6 months after acute ischemic stroke. PCSK9i, proprotein convertase subtilisin/kexin type 9 inhibitor; CI, confidence interval [7,10,15,16,17].

The pooled analysis demonstrated a significant reduction in END with early administration of PCSK9is compared with SOC (OR: 0.39; 95% CI: 0.26–0.57, *p* < 0.00001; I^2^ = 0%; Figure 4). Stroke and/or TIA recurrence and all-cause mortality within 6 months of stroke onset (assessed at study-specific timepoints; Table 3) were also significantly reduced in the PCSK9i group (OR: 0.47; 95% CI: 0.28–0.77, *p* = 0.003; I^2^ = 0% and OR: 0.33; 95% CI: 0.15–0.72, *p* = 0.006; I^2^ = 0%, respectively; Figure 5 and Figure 6).

### 3.7. Secondary Outcomes

#### 3.7.1. Changes in mRS Score

**Figure 7:**
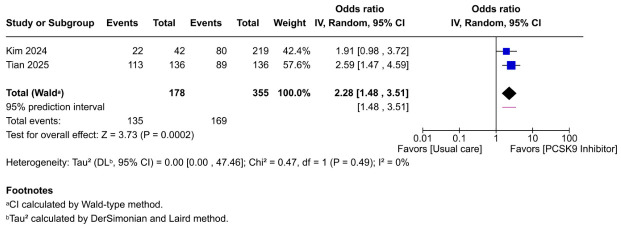
Forest plot of good functional outcomes (modified Rankin Scale [mRS] score of 0–2) at 90 days. Random-effects meta-analysis assessing the likelihood of achieving good functional outcomes, defined as an mRS score of 0–2 at 90 days, among patients receiving early PCSK9i therapy compared with usual care. PCSK9i, proprotein convertase subtilisin/kexin type 9 inhibitor; CI, confidence interval [7,10].

Rates of good functional outcomes at day 90 were significantly higher among patients receiving early PCSK9is versus those receiving SOC (OR: 2.28; 95% CI: 1.48–3.51, *p* = 0.0002; I^2^ = 0%; Figure 7).

#### 3.7.2. Changes in LDL-C Levels

**Figure 8:**
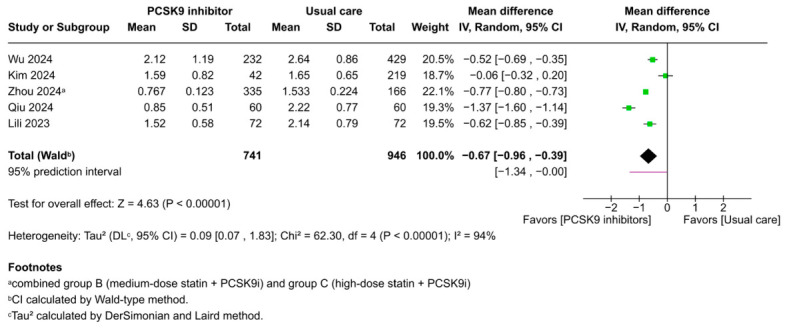
Forest plot of changes in low-density lipoprotein cholesterol (LDL-C) levels. Random-effects meta-analysis showing the effect of early PCSK9i therapy on changes in LDL-C levels compared with usual care. PCSK9i, proprotein convertase subtilisin/kexin type 9 inhibitor; SD, standard deviation; CI, confidence interval [9,10,15,16,18].

PCSK9i use was associated with significantly lower LDL-C levels than SOC was, although with high heterogeneity (MD −0.67 mmol/L; 95% CI −0.96 to −0.39; I^2^ = 94%; Figure 8). For the three-arm study (Zhou et al. [16]), the two PCSK9i arms were combined into a single intervention group, compared against the shared control.

#### 3.7.3. Sensitivity Analysis

Sensitivity analyses based on study design and treatment initiation timing did not alter the statistical significance of the pooled estimates for END (Figure S1), all-cause mortality (Figure S2a,b), or stroke recurrence (Figure S3a,b). Additionally, sensitivity analysis excluding converted LDL-C values in Zhou et al. [16] yielded consistent results (Figure S4).

#### 3.7.4. AEs

Several AEs were reported across five studies (Table 5). Notably, the overall incidence and type of AEs observed with PCSK9i use were comparable to or lower than those with statin monotherapy. This suggests an acceptable short-term tolerability profile for early PCSK9i added to standard lipid-lowering therapy; however, because AEs were secondary outcomes reported over relatively short follow-up in studies not designed to evaluate safety, the certainty of these findings is limited.

## 4. Discussion

Initiating PCSK9i therapy within 3 weeks of AIS onset was associated with reduced rates of END and improved functional outcomes across studies [6,7,18]. Early treatment showed an acceptable short-term tolerability, with most AEs being mild and transient, mainly liver enzyme elevations and injection-site reactions, whereas serious AEs were infrequent and comparable with those observed in control groups [7,15,16].

Combination therapy of PCSK9is and statins has demonstrated favorable clinical outcomes in high-risk populations, irrespective of the initiation timing [19,20,21]. In the ODYSSEY OUTCOMES trial, alirocumab combined with intensive statin therapy after ACS reduced subsequent stroke risk [21]. Similarly, in the FOURIER trial, evolocumab significantly reduced stroke risk, both as an individual event and as part of composite cardiovascular outcomes, although it did not significantly affect all-cause mortality. Only 19.4% of patients had a history of non-hemorrhagic stroke in the FOURIER trial, with a median time of 3.3 years from the most recent event, reflecting a population with predominantly stable and chronic atherosclerosis. Conversely, our study focused on treatment initiation during the acute phase of stroke, which may have contributed to the differences in all-cause mortality between our findings and those reported in the trial [19,20]. Addition of evolocumab as a primary prevention in high-risk patients without known significant atherosclerosis also reduces the risk of major cardiovascular events, including ischemic stroke, over 5 years when compared with placebo [22].

Regarding early administration strategies, a meta-analysis by Hosseini et al. [23] in patients with ACS demonstrated that PCSK9i use reduced major adverse clinical events among patients at cardiovascular risk, although the trends toward reduced stroke/TIA and all-cause mortality were non-significant. The absence of significance might be attributable to the relatively short follow-up duration of 6–18 months [23]. In contrast, the present review showed a significant reduction in early recurrent stroke/TIA [7,9,10,15,16,17] and all-cause mortality within 6 months of PCSK9i therapy [7,10,15,16,17]. This discrepancy could be explained by differences in study populations.

PCSK9 inhibition prevents hepatic LDL-C receptor degradation, resulting in an approximate 50–70% reduction in LDL-C levels [20,24]. A Cochrane review involving more than 67,000 participants similarly reported an average LDL-C reduction of ~55% [20,25]. Consistent with these findings [20,24,25], the present meta-analysis also demonstrated a significant LDL-C reduction, though accompanied by significant heterogeneity.

END affects 5–40% of patients and is associated with poorer functional outcomes and higher mortality [7]. Numerous independent risk factors for END have been identified, including elevated LDL-C levels. Although the pathophysiological mechanisms underlying END remain intricate and not well-understood, unstable atherosclerotic plaque and post-ischemic inflammatory responses are thought to significantly contribute to the occurrence of END [6,7,18]. The role of PCSK9is extends beyond lipid regulation and might be attributed indirectly to their pleiotropic effects, including plaque stabilization, anti-inflammatory properties, and enhancement of endothelial cell function, which address key pathological processes involved in cerebral ischemia development [20,26,27]. These mechanisms may explain the observed improvements in functional outcomes [7,10] and prevention of END in our study [6,7,10,18].

The ischemic penumbra, representing salvageable brain tissue surrounding the infarct core, is particularly vulnerable in the early phase after stroke. Early intervention is therefore critical before progression to the chronic stage, when glial scarring and structural remodeling predominate [28,29]. Within a few hours of a cardiovascular event, PCSK9 levels rise rapidly, contributing to atherosclerotic plaque destabilization [7]. Consequently, early implementation of PCSK9i therapy in patients with ACS has been recognized as beneficial for achieving plaque stabilization and regression [7,23,30]. Similarly, preclinical studies have demonstrated that PCSK9 upregulation in ischemic brain tissue is associated with increased neuronal apoptosis and worse histological outcomes [31]. In AIS, particularly when caused by large artery atherosclerosis, unstable plaques and a high lipid burden pose significant risks for recurrence [9]. As AIS and ACS share similar underlying vascular mechanisms, insights into early treatment may help guide decisions on optimal treatment windows for patients with AIS [7].

### 4.1. Impact of PCSK9i on Intracranial Atherosclerotic Disease

Although the present study focused primarily on clinical outcomes following early administration of PCSK9is, their potential efficacy on intracranial atherosclerotic disease (ICAD) merits specific consideration [32]. ICAD is one of the most common causes of ischemic stroke, with a high risk of recurrence estimated at 10–24% annually [27,33,34].

The effects of PCSK9i use on ICAD were shown by Wu et al. [35], where 49 participants in the PCSK9i group showed improvements on high-resolution magnetic resonance imaging (MRI), including in the degree of stenosis (65.6% vs. 74.2%) and normalized wall index (0.83 vs. 0.86). Moreover, Xiao et al. [27] showed reductions in LDL-C levels at a 6-month follow-up, with a 64% improvement from baseline in the PCSK9i add-on group compared with a statin-only group. Similar to the study by Wu et al. [35], PCSK9i use significantly reduced the degree of stenosis (−11.7) and the plaque enhancement ratio (−13.7) observed on high-resolution MRI [27]. A secondary analysis of the SAMMPRIS trial showed that a 10-mg/dL reduction in LDL-C equaled a 9% lower chance of recurrent stroke or myocardial infarction. Presuming that half of the SAMMPRIS trial population was included and surmising an average projected effect of PCSK9i, there was a 33.2% reduction in the recurrence of stroke or myocardial infarction [32]. There are many ongoing clinical trials researching the efficacy and safety of PCSK9is in AIS and ICAD, demonstrating their potential benefits [36,37,38].

### 4.2. Limitations

This review has certain limitations that should be considered when interpreting the results. Given the observational design of most included studies, the findings may be prone to bias and may limit the depth of analysis. The lack of individual patient data precluded detailed subgroup analyses based on stroke subtype, baseline severity, and lipid levels. Although most included patients had large-artery atherosclerotic stroke, a minority had cardioembolic or other stroke subtypes, and the patient-level data available were insufficient to conduct sensitivity analyses by etiology. The findings, therefore, should be applied primarily to atherosclerotic strokes.

Furthermore, in all included studies, PCSK9is were administered as an adjunct to background statin and/or ezetimibe therapy, and the timing of their initiation was not uniformly reported, so the pooled estimates cannot reliably isolate the independent effect of PCSK9is on the observed outcomes. Most of the included studies excluded patients who had used PCSK9i before the index stroke. Some defined a washout period [7,9], while others excluded any earlier use without specifying a timeframe [10,15,17]. Treatment in these cohorts was therefore initiated de novo, and the pooled population was largely PCSK9i-naïve. Three studies, Lei et al. [6], Lili et al. [18], and Zhou et al. [16], did not report this criterion, so a degree of uncertainty about prior exposure remains for those cohorts.

Although most outcomes had low statistical heterogeneity, baseline characteristics, such as stroke mechanism, baseline NIHSS score, use of reperfusion therapy, and follow-up duration, varied considerably. Additionally, studies assessed outcomes at different timepoints, which may have introduced further heterogeneity into the pooled estimates. PCSK9i therapy lasted only one to three months in some studies. The pooled recurrence and mortality estimates therefore reflect events reported up to each study’s own ascertainment point, not a sustained drug effect maintained throughout a full six-month period. Changes in LDL-C levels showed significant heterogeneity, which could be attributed to different dosing strategies and background lipid-lowering therapies.

The analysis included a relatively limited number of studies and total events for several outcomes, and the corresponding Grading of Recommendations, Assessment, Development, and Evaluations (GRADE) certainty ranged from low to moderate for most outcomes (Table S2). This limitation may have affected the robustness of the analyses and restricted the assessment of publication bias. Moreover, although all studies defined early administration of PCSK9is as occurring within 3 weeks of stroke onset, there was considerable variation in the specific timing of initiation, and the optimal timing remains undefined. The relatively short follow-up duration in most studies further limits the ability to determine the long-term efficacy and safety of early PCSK9i initiation.

Finally, all included studies were conducted in Asian (predominantly Chinese) populations, potentially limiting the generalizability of the findings to other populations and healthcare settings. The economic implications of early PCSK9i use should also be considered. PCSK9is are more costly than statins, and acquisition costs are considerably higher in many countries than in Asian countries, where most included studies were conducted. Therefore, the cost-effectiveness of PCSK9is in the acute stroke setting may need to be analyzed separately for different healthcare systems.

## 5. Conclusions

In patients with predominantly atherosclerotic AIS, initiating PCSK9i therapy within 21 days of stroke onset was associated with reduced risk of END, recurrent stroke/TIA, and mortality, with an acceptable short-term tolerability. Further large-scale trials are needed in diverse populations, including non-Asian populations, to clearly establish the optimal timing of administration and to assess long-term efficacy, safety, and cost-effectiveness.

## Figures and Tables

**Table 1:** Characteristics of the included studies.

Study	Country	Study Design	Age (y), Mean ± SD/Median (Range)	Number of Patients	Male, *n* (%)	Type of Stroke	Stroke Onset
Tian et al. [7]	China	RCT	PCSK9i: 63.61 ± 10.08, Control: 65.87 ± 8.88	272	196 (72.06%)	Atherosclerotic origin (LAA and SVO)	≤24 h
Qiu et al. [15]	China	RCT	PCSK9i: 60.97 ± 7.17, Control: 62.88 ± 9.13	120	84 (70%)	Atherosclerotic origin (unspecified)	≤24 h
Zhou et al. [16]	China	RCT	Medium-dose statin + PCSK9i: 65.6 ± 11.3 High-dose statin + PCSK9i: 64.9 ± 11.7 Control: 65.2 ± 12.6	501	305 (60.9%)	Atherosclerotic origin (unspecified)	≤72 h
Wu et al. [9]	China	Prospective cohort	66.26 ± 12.20	661	414 (62.63%)	Atherosclerotic origin (LAA)	≤1 wk
Lei et al. [6]	China	Retrospective case–control	63.344 ± 12.901	250	56 (22.4%)	Atherosclerotic origin (LAA, SVO, SOE, SUE)	≤72 h
Kim et al. [10]	South Korea	Retrospective cohort	69.2 ± 11.7	261	112 (42.9%)	Atherosclerotic (LAA and others) and cardioembolic origin	≤24 h
Zhang et al. [17]	China	Retrospective	PCSK9i: 63 (56.0–70.0) Control: 64.5 (57.0–71.0)	528	374 (70.83%)	Atherosclerotic origin (LAA, SVO, undetermined)	≤14 d
Lili et al. [18]	China	Retrospective cohort	61.2 ± 9.6	144	90 (62.5%)	Atherosclerotic origin (LAA)	≤72 h

Abbreviations: RCT, randomized controlled trial; PCSK9i, proprotein convertase subtilisin/kexin type 9 inhibitor; LAA, large artery atherosclerosis; SVO, small-vessel occlusion; SOE, stroke of other etiology; SUE, stroke of undetermined etiology; SD, standard deviation.

**Table 2:** Patient baseline characteristics.

Study	Arms	HTN	DM	CAD	Prior Ischemic Stroke	LDL-C Baseline Level (mmol/L) ^a^	Lp(a) Baseline Level (mmol/L) ^a^	Baseline mRS Score	Baseline NIHSS Score, Mean ± SD/Median (Range/IQR/Q1, Q3) ^a^
Tian et al. [7]	Statins	89 (65.4%)	47 (34.6%)	18 (13.2%)	30 (22.1%)	2.83 ± 0.69	N/A	≤1	3 (1, 4) NIHSS score ≤ 4: 103 (75.7%) NIHSS score > 4: 33 (24.3%)
	Evolocumab + Statins	97 (71.3%)	58 (42.6%)	18 (13.2%)	30 (22.1%)	2.76 ± 0.82	N/A	≤1	3 (1, 5) NIHSS score ≤ 4: 96 (70.6%) NIHSS score > 4: 40 (29.4%)
Qiu et al. [15]	SOC	40 (66.67%)	22 (36.67%)	6 (10.0%)	19 (31.67%)	3.17 ± 0.83	72.93 ± 96.86	≤2	2.25 ± 1.51
	Evolocumab	47 (78.33%)	21 (35.0%)	4 (6.67%)	21 (35%)	3.15 ± 0.96	88.40 ± 117.30	≤2	3.28 ± 3.07
Zhou et al. [16]	Statin	101 (60.8%)	31 (18.7%)	13 (7.8%)	26 (15.7%)	3.1 (2.7, 3.4)	185.5 (97.7, 358.0)	<1	3 (1, 5)
	Statins 10 mg + Alirocumab	107 (64.1%)	41 (24.6%)	22 (13.2%)	15 (9.0%)	2.9 (2.8,3.4)	136 (69.0, 319.0)	<1	2 (0, 5)
	Statins 20 mg + Alirocumab	109 (64.9%)	40 (23.8%)	19 (11.3%)	20 (11.9%)	2.9 (2.7,3.4)	161.0 (69.5, 336.5)	<1	3 (0, 6)
Wu et al. [9]	Control	321 (74.83%)	176 (41.03%)	43 (10.02%)	103 (24.01%)	2.91 ± 1.05	298.96 ± 280.19	0–2: 101 (23.54%)	3.40 ± 4.59
	PCSK9is	159 (68.53%)	82 (35.34%)	21 (9.05%)	70 (30.17%)	3.06 ± 1.16	283.44 ± 303.52	0–2: 51 (21.98%)	3.03 ± 2.94
Lei et al. [6]	PCSK9is	169 (67.6%)	74 (29.60%)	44 (17.60%)	81 (32.40%)	2.760 (2.070, 3.400)	N/A	N/A	3(1–7)
Kim et al. [10]	Control	134 (61.2%)	63 (28.8%)	N/A	34 (15.5%)	2.46 ± 0.98	N/A	0: 185 (84.5%), 1: 16 (7.3%), 2: 18 (8.2%)	16 (11, 19)
	Evolocumab	28 (66.7%)	11 (26.2%)	N/A	7 (16.7%)	2.61 ± 1.02	N/A	0: 36 (85.7%), 1: 4 (9.5%), 2: 2 (4.8%)	14 (10.25, 18.75)
Zhang et al. [17]	SOC	237 (73.6%)	109 (33.9%)	86 (26.7%)	121 (37.6%)	2.696 (2.48–3.14)	N/A	N/A	NIHSS score ≤ 8: 253 (78.6%) NIHSS score 9– ≤ 15: 58 (18%) NIHSS score ≥16: 11 (3.4%)
	Evolocumab	148 (71.8%)	77 (37.4%)	51 (24.8%)	70 (34%)	3.55 (2.94–4.34)	N/A	N/A	NIHSS score ≤ 8: 150 (72.8%) NIHSS score 9–15: 40 (19.4%) NIHSS score ≥ 16: 16 (7.8%)
Lili et al. [18]	Alirocumab	48 (66.7%)	32 (44.4%)	N/A	N/A	3.00 ± 0.82	283 ± 54 mg/L	N/A	3 (1, 4)
	Statins	53 (73.6%)	33 (45.8%)	N/A	N/A	3.12 ± 0.91	286 ± 45 mg/L	N/A	3 (1, 4)

Abbreviations: HTN, hypertension; DM, diabetes mellitus; CAD, coronary artery disease; LDL-C, low-density lipoprotein cholesterol; Lp(a), lipoprotein A; mRS, modified Rankin scale; NIHSS, National Institutes of Health Stroke Scale; N/A, not available; SOC, standard of care; PCSK9is, proprotein convertase subtilisin/kexin type 9 inhibitor. ^a^ Data are presented as mean ± SD or median (interquartile range).

**Table 3:** Intervention characteristics of the included studies.

Study	Intervention (Dose, Frequency)	Adjunctive Lipid-Lowering Treatment	Duration of Treatment	Control (Dose)	IVT or EVT	Outcome Measures (Timepoints)
Tian et al. [7]	Evolocumab (140 mg, Q2W)	Atorvastatin	90 d	Atorvastatin (40 mg)	None	- END (≤24 h–7 days) - LDL-C (d 7) - Mortality (≤7 d) - mRS score ≤2 (d 90) - Stroke recurrence (≤90 d) - AEs (baseline, d 7, and d 30)
Qiu et al. [15]	Evolocumab (240 mg, Q4W)	Atorvastatin and ezetimibe	8 wk	Atorvastatin and Ezetimibe (40 mg and 10 mg)	None	- LDL-C (wk 8) - Stroke recurrence (wk 8) - Mortality (wk 8) - AEs (baseline to wk 8)
Zhou et al. [16]	Alirocumab (75 mg, Q2W)	Rosuvastatin	90 d	Rosuvastatin (20 mg)	None	- LDL-C (d 90) - Stroke recurrence (≤90 d) - mRS score ≤2 (d 90) - Liver dysfunction (≤90 d) - Mortality (≤90 d) - AEs (90 d)
Wu et al. [9]	Alirocumab (75 mg, Q2W) or evolocumab (140 mg or 420 mg, Q2W)	Statins and/or ezetimibe	30 d	Statins and/or ezetimibe (10–20 mg)	None	- LDL-C (baseline and d 30) - Stroke recurrence (≤30 d)
Lei et al. [6]	PCSK9is (unspecified)	None	N/A	N/A	IVT	- END (≤7 d)
Kim et al. [10]	Evolocumab (140 mg)	None	N/A	N/A	EVT	- END (≤7 d) - Mortality (≤90 d) - mRS score ≤2 (d 90) - Stroke recurrence (≤90 d) - LDL-C (baseline and d 90) - AEs (N/A)
Zhang et al. [17]	Evolocumab (140 mg, Q2W)	Statins or SOC	12 months	N/A	None	- Lipid profile (baseline and mo 12) - Stroke recurrence (6 mo) - Mortality (6 mo) - AEs (N/A)
Lili et al. [18]	Alirocumab (75 mg, single dose)	Statin	N/A	N/A	None	- LDL-C level (baseline and d 3) - END (≤72 h)

Abbreviations: IVT, intravenous thrombolysis; EVT, endovascular therapy; END, early neurological deterioration; LDL-C, low-density lipoprotein cholesterol; mRS, modified Rankin scale; AEs, adverse events; PCSK9is, proprotein convertase subtilisin/kexin type 9 inhibitor; N/A, not available; SOC, standard of care.

**Table 4:** Quality assessment of observational studies.

Cohort Studies									
Study	Selection				Comparability	Outcome			Total Score
	Representativeness of the Exposed Cohort	Selection of the Non-Exposed Cohort	Ascertainment of Exposure	Demonstration That Outcome of Interest Was Not Present at Start of Study	Comparability of Cohorts on the Basis of the Design or Analysis	Assessment of Outcome	Was the Follow-Up Long Enough for Outcomes to Occur?	Adequacy of Follow-Up of Cohorts	
Wu et al. [9]	*	*	*	*	*	*	*	*	8/9 (good)
Kim et al. [10]	*	*	*	*	**	*	*		8/9 (good)
Zhang et al. [17]		*	*	*	**	*	*	*	8/9 (good)
Lili et al. [18]	*	*	*	*	**	*	*		8/9 (good)
**Case Control Studies**									
Study	Selection				Comparability	Exposure			Total score
	Is the case definition adequate?	Representativeness of the cases	Selection of controls	Definition of controls	Comparability of cases and controls on the basis of the design or analysis controlled for confounders	Ascertainment of exposure	Same method of ascertainment for cases and controls	Non-response rate	
Lei et al. [6]	*	*	*	*	*	*	*	*	8/9 (good)

* Indicates one star awarded according to the Newcastle–Ottawa Scale (NOS). ** Indicates two stars awarded for the comparability domain (maximum possible score for this domain).

**Table 5:** Adverse events.

Study	Adverse Event	PCSK9i *n*/*N* (%)	Control *n*/*N* (%)
Tian et al. [7]	Mild liver or kidney laboratory abnormalities	15/136 (11%)	22/136 (16.2%)
	Bleeding	0	0
	Infection	0	0
	Injection-site reaction	0	0
	Treatment discontinuation due to AE	0	0
Qiu et al. [15]	Any adverse event	5/60 (8.3%)	6/60 (10%)
	Elevated liver enzymes (ALT/AST > 3 × ULN)	2/60 (3.3%)	2/60 (3.3%)
	Injection-site reaction	2/60 (3.3%)	0/60 (0%)
	Neurocognitive events	1/60 (1.7%)	1/60 (1.7%)
	Cerebral hemorrhage	0/60 (0%)	1/60 (1.7%)
Zhou et al. [16]	Hepatic insufficiency (transaminase elevation ≥ 3 × normal)	Low dose: 1/167 (0.6%); High dose: 9/168 (5.4%)	10/166 (6%)
Kim et al. [10]	Hemorrhagic events	7/42 (16.7%)	74/219 (33.8%)
	Symptomatic intracerebral hemorrhage	1/42 (2.4%)	19/219 (8.7%)
Zhang et al. [17]	Allergic reactions	8/206 (3.9%)	8/322 (2.5%)
	Injection-site reactions	3/206 (1.5%)	0
	Neurocognitive events	3/206 (1.5%)	4/322 (1.2%)
	Musculoskeletal pain	4/206 (1.9%)	6/322 (1.8%)
	New-onset diabetes	7/206 (3.4%)	6/322 (1.8%)

PCSK9i, proprotein convertase subtilisin/kexin type 9 inhibitor; AE, adverse event; ALT, alanine transaminase; AST, aspartate transaminase; ULN, upper limit of normal.

## Data Availability

The data will be made available upon reasonable request from the corresponding author.

## Supplementary Materials

The following supporting information can be downloaded at: https://www.mdpi.com/article/10.3390/jcm15135169/s1, Table S1: Search strategy; Figure S1: Subgroup analysis of END based on study design; Figure S2a: Subgroup analysis of all-cause mortality ≤6 months based on timing; Figure S2b: Subgroup analysis of all-cause mortality ≤6 months based on study design; Figure S3a: Subgroup analysis of recurrent stroke/TIA ≤6 months based on timing; Figure S3b: Subgroup analysis of recurrent stroke/TIA ≤6 months based on study design; Figure S4: Subgroup analysis of LDL-C level changes, excluding converted data; Table S2: Certainty (GRADE) Assessment; Table S3: PRISMA 2020 Checklist [39]; Table S4: PRISMA 2020 for Abstracts Checklist [39].

## Abbreviations

The following abbreviations are used in this manuscript:

ACS	acute coronary syndrome
AE	adverse event
AIS	acute ischemic stroke
ALT	alanine aminotransferase
AST	aspartate aminotransferase
CAD	coronary artery disease
CI	confidence interval
CNS	central nervous system
DM	diabetes mellitus
END	early neurological deterioration
EVT	endovascular therapy
GRADE	Grading of Recommendations, Assessment, Development, and Evaluations
HTN	Hypertension
ICAD	intracranial atherosclerotic disease
IQR	interquartile range
IVT	intravenous thrombolysis
LAA	large artery atherosclerosis
LDL-C	low-density lipoprotein cholesterol
Lp(a)	lipoprotein(a)
MD	mean difference
MRI	magnetic resonance imaging
mRS	modified Rankin Scale
NIHSS	National Institutes of Health Stroke Scale
OR	odds ratio
PCSK9	proprotein convertase subtilisin/kexin type 9
PCSK9i	proprotein convertase subtilisin/kexin type 9 inhibitor
PRISMA	Preferred Reporting Items for Systematic Reviews and Meta-Analyses
Q2W	every 2 weeks
Q4W	every 4 weeks
RCT	randomized controlled trial
RevMan	Review Manager
RoB	risk of bias
SD	standard deviation
SOC	standard of care
SOE	stroke of other etiology
SUE	stroke of undetermined etiology
SVO	small-vessel occlusion
TIA	transient ischemic attack
ULN	upper limit of normal
