# Supplementary material for: Clinical Outcomes of Early Administration of Proprotein Convertase Subtilisin/Kexin Type 9 Inhibitors in East Asian Patients with Acute Ischemic Stroke: A Systematic Review and Meta-Analysis
Source: J Clin Med. 2026 Jul 2;15(13):5169. doi: 10.3390/jcm15135169 (PMC13362794; doi:10.3390/jcm15135169)
Supplement: Supplementary file 1 [file jcm-15-05169-s001.zip › jcm-4365752-supplementary.pdf]

Supplementary Materials

Table S1. Search strategy

Figure S1. Subgroup analysis of END based on study design

Figure S2a. Subgroup analysis of all-cause mortality ≤6 months based on timing

Figure S2b. Subgroup analysis of all-cause mortality ≤6 months based on study design

Figure S3a. Subgroup analysis of recurrent stroke/TIA ≤6 months based on timing

Figure S3b. Subgroup analysis of recurrent stroke/TIA ≤6 months based on study design

Figure S4. Subgroup analysis of LDL-C level changes, excluding converted data

Table S2. Certainty (GRADE) Assessment

Table S3. PRISMA 2020 Checklist

Table S4. PRISMA 2020 for Abstracts Checklist

Table S1. Search strategy

PubMed	(("PCSK9 Inhibitors"[Mesh]) OR (PCSK9 inhibitor OR evolocumab OR alirocumab)) AND (("Ischemic Stroke"[Mesh]) OR (ischemic stroke OR cerebrovascular event OR brain infarction))
Web of Science—All fields	("PCSK9 inhibitor*" OR "evolocumab" OR "alirocumab") AND ("ischemic stroke" OR "cerebrovascular event" OR "brain infarction")
Ovid-Embase	((PCSK9 Inhibitors OR PCSK9 inhibitor OR evolocumab OR alirocumab) AND (ischemic stroke))
Cochrane library	((PCSK9 Inhibitor*) OR (evolocumab) OR (alirocumab) AND (ischemic stroke))
Google scholar	allintitle: (ischemic stroke) (PCSK9 inhibitor) OR (PCSK9 Inhibitors) OR (evolocumab) OR (alirocumab)
SCOPUS	((PCSK9 Inhibitors) OR (PCSK9 inhibitor OR evolocumab OR alirocumab) AND (ischemic stroke OR cerebrovascular event OR brain infarction))
MEDLINE	((PCSK9 Inhibitor* OR evolocumab OR alirocumab) AND (ischemic stroke))
VHL—all https://bvsalud.org/en/	((PCSK9 Inhibitor*) OR (evolocumab) OR (alirocumab) AND (ischemic stroke))

Study or Subgroup	PCSK9 Inhibitor Events	PCSK9 Inhibitor Total	Usual care Events	Usual care Total	Weight	Odds ratio IV, Random, 95% CI
**1.1.1 RCTs**						
Tian 2025	18	136	33	136	36.9%	0.48 [0.25 , 0.90]
**Subtotal**		**136**		**136**	**36.9%**	**0.48 [0.25 , 0.90]**
Total events:	18		33			
Test for overall effect: Z = 2.30 (P = 0.02)						
Heterogeneity: Not applicable						
**1.1.2 Observational studies**						
Kim 2024	5	42	44	219	15.0%	0.54 [0.20 , 1.45]
Lei 2024	12	135	29	115	27.9%	0.29 [0.14 , 0.60]
Lili, 2023	9	72	23	72	20.1%	0.30 [0.13 , 0.72]
**Subtotal (Wald[a])**		**249**		**406**	**63.1%**	**0.34 [0.21 , 0.55]**
Total events:	26		96			
Test for overall effect: Z = 4.36 (P < 0.0001)						
Heterogeneity: Tau² (DL[b], 95% CI) = 0.00 [0.00 , 4.45]; Chi² = 1.07, df = 2 (P = 0.58); I² = 0%						
**Total (Wald[a])**		**385**		**542**	**100.0%**	**0.39 [0.26 , 0.57]**
95% prediction interval						[0.26 , 0.57]
Total events:	44		129			
Test for overall effect: Z = 4.86 (P < 0.00001)						
Test for subgroup differences: Chi² = 0.68, df = 1 (P = 0.41), I² = 0%						
Heterogeneity: Tau² (DL[b], 95% CI) = 0.00 [0.00 , 1.17]; Chi² = 1.75, df = 3 (P = 0.63); I² = 0%						

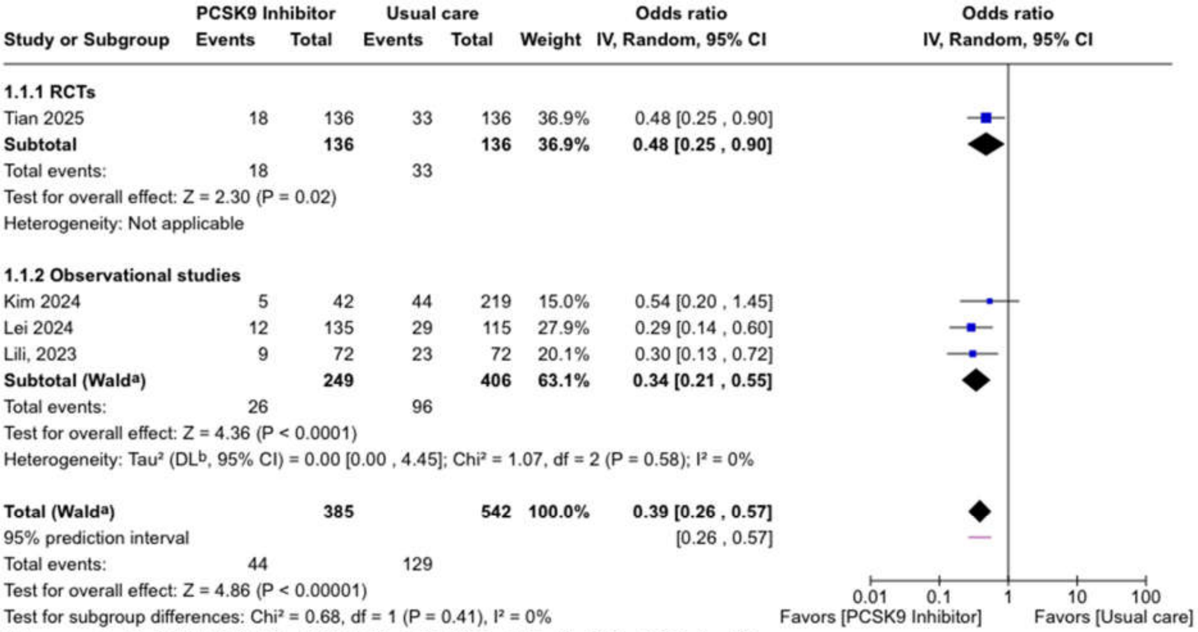

Footnotes

[a]CI calculated by Wald-type method.

[b]Tau² calculated by DerSimonian and Laird method.

Figure S1. Subgroup analysis of END based on study design

Study or Subgroup	PCSK9 Inhibitor Events	PCSK9 Inhibitor Total	Usual care Events	Usual care Total	Weight	Odds ratio IV, Random, 95% CI
**2.1.1 ≤ 72 hours**						
Kim 2024	3	42	39	219	40.6%	0.36 [0.10 , 1.21]
Qiu 2024	0	60	0	60		Not estimable
Tian 2025	0	136	2	136	6.6%	0.20 [0.01 , 4.14]
Zhou 2024	3	335	4	166	26.7%	0.37 [0.08 , 1.65]
**Subtotal (Wald[a])**		**573**		**581**	**73.9%**	**0.34 [0.14 , 0.84]**
Total events:	6		45			
Test for overall effect: Z = 2.33 (P = 0.02)						
Heterogeneity: Tau² (DL[b], 95% CI) = 0.00 [0.00 , 3.03]; Chi² = 0.14, df = 2 (P = 0.93); I² = 0%						
**2.1.2 > 72 hours**						
Zhang 2023	2	206	10	322	26.1%	0.31 [0.07 , 1.41]
**Subtotal**		**206**		**322**	**26.1%**	**0.31 [0.07 , 1.41]**
Total events:	2		10			
Test for overall effect: Z = 1.52 (P = 0.13)						
Heterogeneity: Not applicable						
**Total (Wald[a])**		**779**		**903**	**100.0%**	**0.33 [0.15 , 0.72]**
95% prediction interval						[0.15 , 0.72]
Total events:	8		55			
Test for overall effect: Z = 2.78 (P = 0.006)						
Test for subgroup differences: Chi² = 0.01, df = 1 (P = 0.91), I² = 0%						
Heterogeneity: Tau² (DL[b], 95% CI) = 0.00 [not estimable]; Chi² = 0.15, df = 3 (P = 0.99); I² = 0%						

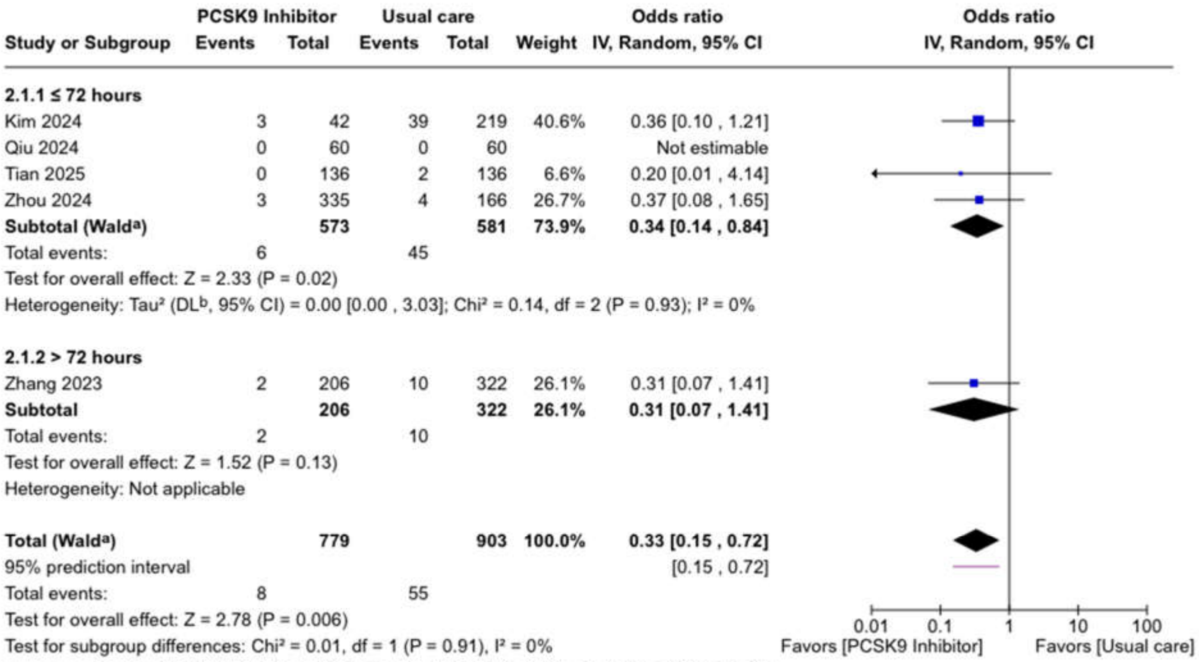

Footnotes

[a]CI calculated by Wald-type method.

[b]Tau² calculated by DerSimonian and Laird method.

Figure S2a. Subgroup analysis of all-cause mortality at ≤6 months based on timing

Study or Subgroup	PCSK9 Inhibitor Events	PCSK9 Inhibitor Total	Usual care Events	Usual care Total	Weight	Odds ratio IV, Random, 95% CI
**2.2.1 RCTs**						
Qiu 2024	0	60	0	60		Not estimable
Tian 2025	0	136	2	136	6.6%	0.20 [0.01 , 4.14]
Zhou 2024	3	335	4	166	26.7%	0.37 [0.08 , 1.65]
**Subtotal (Wald[a])**		**531**		**362**	**33.3%**	**0.32 [0.08 , 1.25]**
Total events:	3		6			
Test for overall effect: Z = 1.63 (P = 0.10)						
Heterogeneity: Tau² (DL[b], 95% CI) = 0.00 [0.00 , >100]; Chi² = 0.13, df = 1 (P = 0.72); I² = 0%						
**2.2.2 Observational studies**						
Kim 2024	3	42	39	219	40.6%	0.36 [0.10 , 1.21]
Zhang 2023	2	206	10	322	26.1%	0.31 [0.07 , 1.41]
**Subtotal (Wald[a])**		**248**		**541**	**66.7%**	**0.33 [0.13 , 0.87]**
Total events:	5		49			
Test for overall effect: Z = 2.24 (P = 0.02)						
Heterogeneity: Tau² (DL[b], 95% CI) = 0.00 [0.00 , 10.80]; Chi² = 0.02, df = 1 (P = 0.88); I² = 0%						
**Total (Wald[a])**		**779**		**903**	**100.0%**	**0.33 [0.15 , 0.72]**
95% prediction interval						[0.15 , 0.72]
Total events:	8		55			
Test for overall effect: Z = 2.78 (P = 0.006)						
Test for subgroup differences: Chi² = 0.00, df = 1 (P = 0.97), I² = 0%						
Heterogeneity: Tau² (DL[b], 95% CI) = 0.00 [not estimable]; Chi² = 0.15, df = 3 (P = 0.99); I² = 0%						

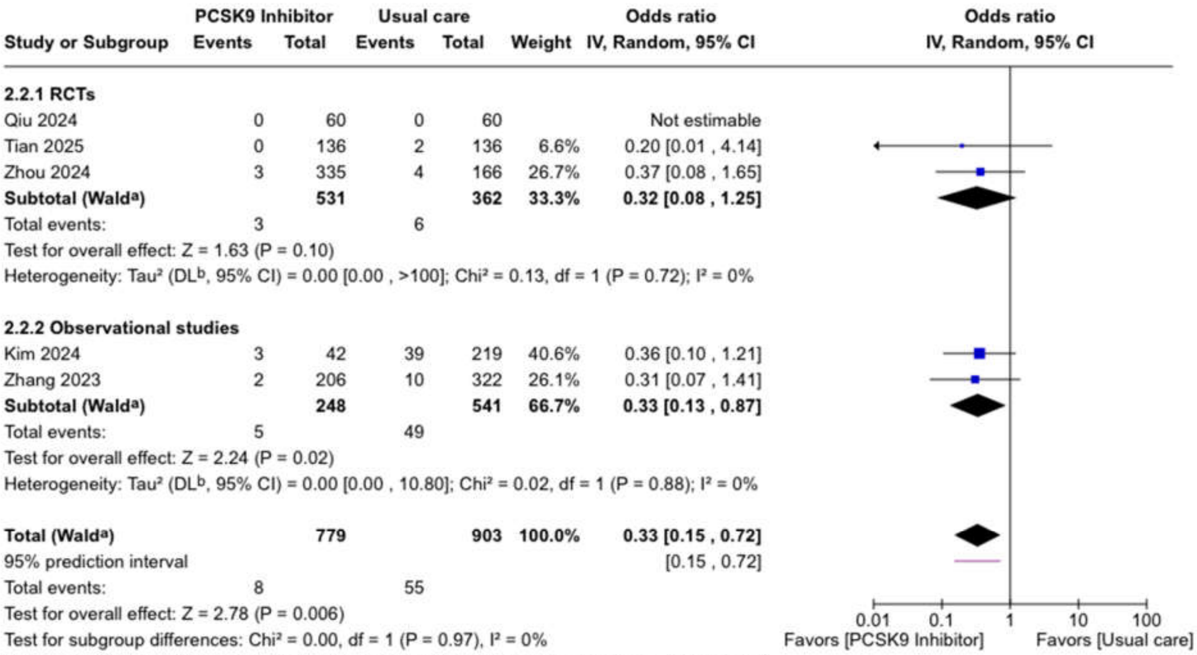

Footnotes

[a]CI calculated by Wald-type method.

[b]Tau² calculated by DerSimonian and Laird method.

Figure S2b. Subgroup analysis of all-cause mortality at ≤6 months based on study design

Study or Subgroup	PCSK9 Inhibitor Events	PCSK9 Inhibitor Total	Usual care Events	Usual care Total	Weight	Odds ratio IV, Random, 95% CI
**3.1.1 ≤ 72 hours**						
Kim 2024	5	42	21	219	23.4%	1.27 [0.45 , 3.59]
Qiu 2024	0	60	1	60	2.4%	0.33 [0.01 , 8.21]
Tian 2025	1	136	4	136	5.2%	0.24 [0.03 , 2.22]
Zhou 2024	9	335	12	166	32.0%	0.35 [0.15 , 0.86]
**Subtotal (Wald[a])**		**573**		**581**	**63.0%**	**0.54 [0.24 , 1.24]**
Total events:	15		38			
Test for overall effect: Z = 1.45 (P = 0.15)						
Heterogeneity: Tau² (DL[b], 95% CI) = 0.19 [0.00 , 6.87]; Chi² = 4.09, df = 3 (P = 0.25); I² = 27%						
**3.1.2 >72 hours**						
Wu 2024	5	232	24	429	26.3%	0.37 [0.14 , 0.99]
Zhang 2023	2	206	10	322	10.7%	0.31 [0.07 , 1.41]
**Subtotal (Wald[a])**		**438**		**751**	**37.0%**	**0.35 [0.15 , 0.80]**
Total events:	7		34			
Test for overall effect: Z = 2.49 (P = 0.01)						
Heterogeneity: Tau² (DL[b], 95% CI) = 0.00 [0.00 , 18.91]; Chi² = 0.04, df = 1 (P = 0.83); I² = 0%						
**Total (Wald[a])**		**1011**		**1332**	**100.0%**	**0.47 [0.28 , 0.77]**
95% prediction interval						[0.28 , 0.77]
Total events:	22		72			
Test for overall effect: Z = 2.98 (P = 0.003)						
Test for subgroup differences: Chi² = 0.54, df = 1 (P = 0.46), I² = 0%						
Heterogeneity: Tau² (DL[b], 95% CI) = 0.00 [0.00 , 1.62]; Chi² = 4.86, df = 5 (P = 0.43); I² = 0%						

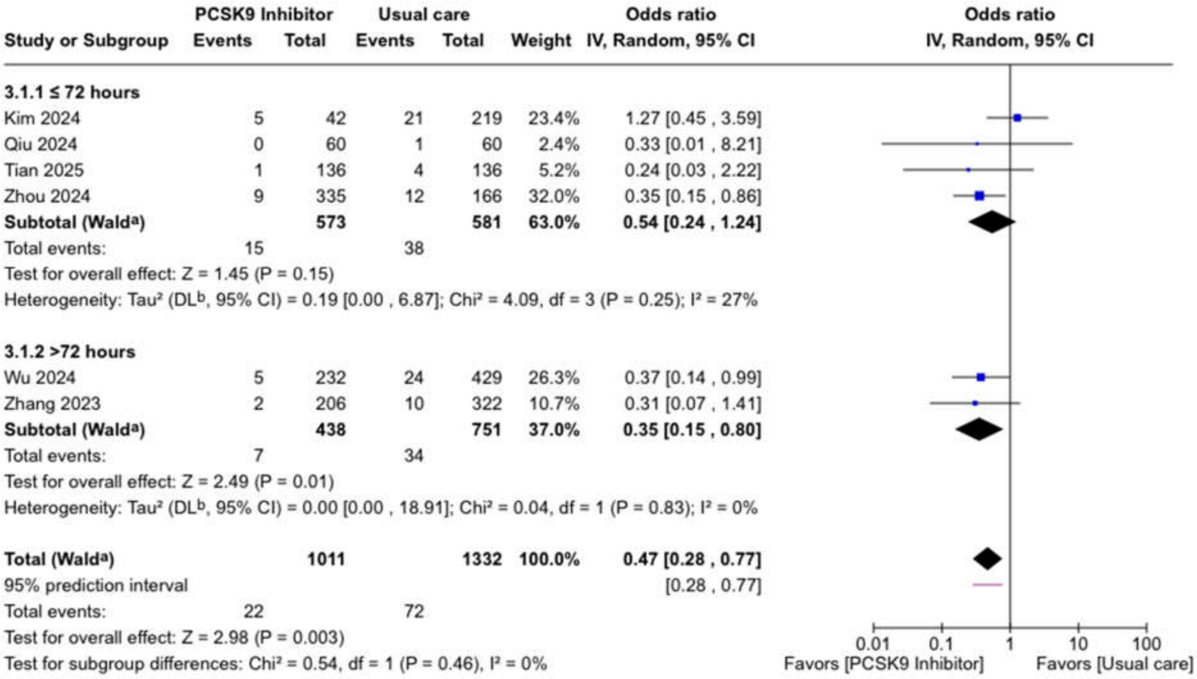

Footnotes

[a]CI calculated by Wald-type method.

[b]Tau² calculated by DerSimonian and Laird method.

Figure S3a. Subgroup analysis of recurrent stroke/TIA at ≤ 6 months based on timing

Study or Subgroup	PCSK9 Inhibitor Events	PCSK9 Inhibitor Total	Usual care Events	Usual care Total	Weight	Odds ratio IV, Random, 95% CI
**3.2.1 RCTs**						
Qiu 2024	0	60	1	60	2.4%	0.33 [0.01 , 8.21]
Tian 2025	1	136	4	136	5.2%	0.24 [0.03 , 2.22]
Zhou 2024	9	335	12	166	32.0%	0.35 [0.15 , 0.86]
**Subtotal (Wald[a])**		**531**		**362**	**39.6%**	**0.34 [0.15 , 0.74]**
Total events:	10		17			
Test for overall effect: Z = 2.69 (P = 0.007)						
Heterogeneity: Tau² (DL[b], 95% CI) = 0.00 [0.00 , 0.63]; Chi² = 0.09, df = 2 (P = 0.95); I² = 0%						
**3.2.2 Observational studies**						
Kim 2024	5	42	21	219	23.4%	1.27 [0.45 , 3.59]
Wu 2024	5	232	24	429	26.3%	0.37 [0.14 , 0.99]
Zhang 2023	2	206	10	322	10.7%	0.31 [0.07 , 1.41]
**Subtotal (Wald[a])**		**480**		**970**	**60.4%**	**0.56 [0.23 , 1.38]**
Total events:	12		55			
Test for overall effect: Z = 1.26 (P = 0.21)						
Heterogeneity: Tau² (DL[b], 95% CI) = 0.29 [0.00 , 23.29]; Chi² = 3.68, df = 2 (P = 0.16); I² = 46%						
**Total (Wald[a])**		**1011**		**1332**	**100.0%**	**0.47 [0.28 , 0.77]**
95% prediction interval						[0.28 , 0.77]
Total events:	22		72			
Test for overall effect: Z = 2.98 (P = 0.003)						
Test for subgroup differences: Chi² = 0.70, df = 1 (P = 0.40), I² = 0%						
Heterogeneity: Tau² (DL[b], 95% CI) = 0.00 [0.00 , 1.62]; Chi² = 4.86, df = 5 (P = 0.43); I² = 0%						

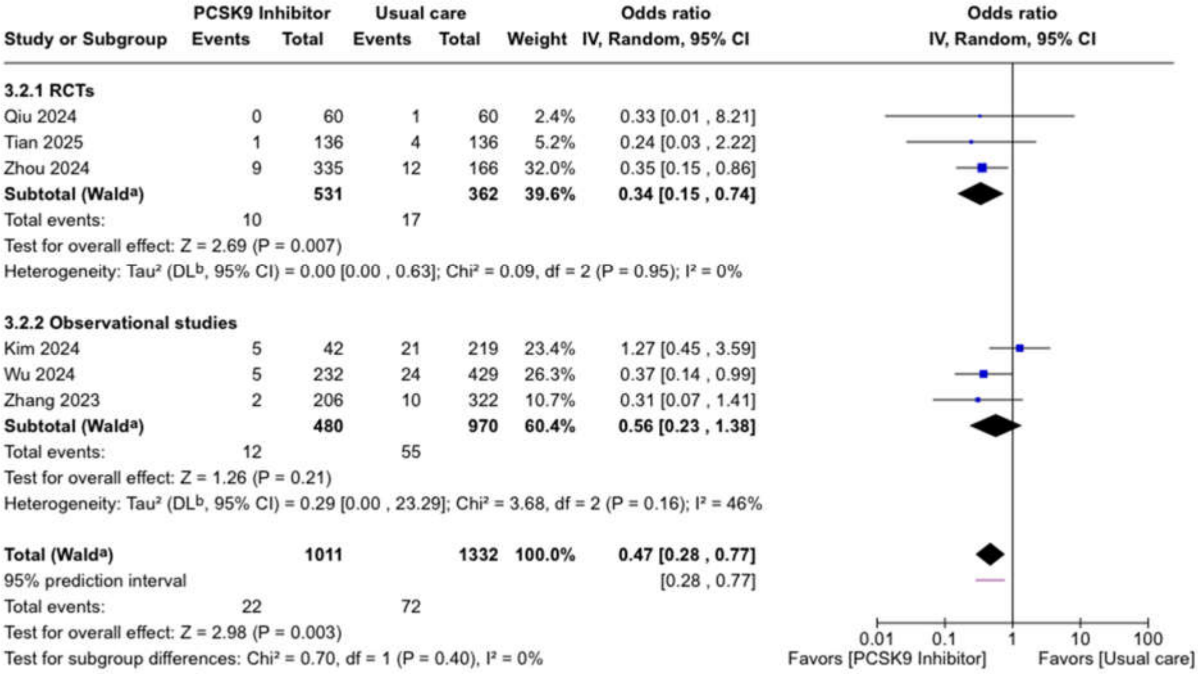

Footnotes

[a]CI calculated by Wald-type method.

[b]Tau² calculated by DerSimonian and Laird method.

Figure S3b. Subgroup analysis of recurrent stroke/TIA at ≤6 months based on study design

Study or Subgroup	Mean	SD	Total	Mean	SD	Total	Weight	Mean difference IV, Random, 95% CI
Kim 2024	1.59	0.82	42	1.65	0.65	219	24.5%	-0.06 [-0.32 , 0.20]
Lili, 2023	1.52	0.58	72	2.14	0.79	72	25.0%	-0.62 [-0.85 , -0.39]
Qiu 2024	0.85	0.51	60	2.22	0.77	60	24.9%	-1.37 [-1.60 , -1.14]
Wu 2024	2.12	1.19	232	2.64	0.86	429	25.6%	-0.52 [-0.69 , -0.35]
**Total (Wald[a])**			**406**			**780**	**100.0%**	**-0.64 [-1.13 , -0.16]**
95% prediction interval								[-1.71 , 0.42]
Test for overall effect: Z = 2.60 (P = 0.009)								
Heterogeneity: Tau² (DL[b], 95% CI) = 0.23 [0.08 , 4.08]; Chi² = 58.09, df = 3 (P < 0.00001); I² = 95%								

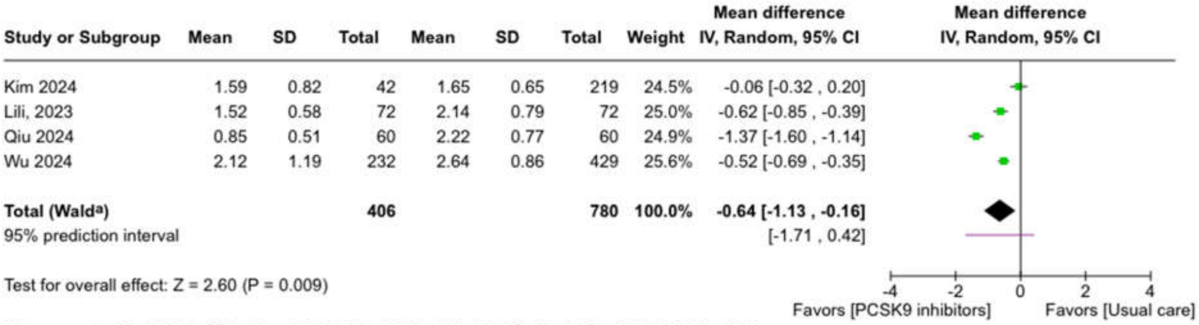

Footnotes

[a]CI calculated by Wald-type method.

[b]Tau² calculated by DerSimonian and Laird method.

Figure S4. Subgroup analysis of LDL-C level changes, excluding converted data

Table S2. Certainty Assessment

Outcome	*No. of studies (Design)*	*Effect*	*Certainty (GRADE)*	*Importance*
Early neurological deterioration (END)	4 (1 RCT, 3 observational)	OR: 0.39; 95% CI: 0.26–0.57).	⊕⊕⊕◯ MODERATE[1], due to risk of bias	Critical
Stroke/TIA recurrence	6(3 RCTs, 3 observational)	OR: 0.47; 95% CI: 0.28–0.77	⊕⊕⊕◯ MODERATE[1], due to risk of bias	Critical
All-cause mortality	5 (3 RCTs, 2 observational)	OR: 0.33; 95% CI: 0.15–0.72	⊕⊕◯◯ LOW[1,2], due to risk of bias and imprecision	Critical
Good functional outcome (mRS 0-2)	2 (1 RCT, 1 observational)	OR: 2.28; 95% CI: 1.48 ‑ 3.51	⊕⊕⊕◯ MODERATE[1], due to risk of bias	Critical
Change in LDL levels	5 (2 RCTs, 3 observational)	MD −0.67 mmol/L; 95% CI −0.96 to −0.39	⊕◯◯◯ VERY LOW[1,3], due to risk of bias and inconsistency	Important

[1]Majority of weight is non-randomized with some concerns in the randomization and selection process

[2]Low event count for all-cause mortality, n=63 across all studies

[3]Heterogeneity is 94%

Table S3. PRISMA 2020 Checklist

Section and Topic	Item #	Checklist item	Location where item is reported
**TITLE**			
Title	1	Identify the report as a systematic review.	P1
**ABSTRACT**			
Abstract	2	See the PRISMA 2020 for Abstracts checklist.	Table S4
**INTRODUCTION**			
Rationale	3	Describe the rationale for the review in the context of existing knowledge.	P2
Objectives	4	Provide an explicit statement of the objective(s) or question(s) the review addresses.	P2
**METHODS**			
Eligibility criteria	5	Specify the inclusion and exclusion criteria for the review and how studies were grouped for the syntheses.	P2-3
Information sources	6	Specify all databases, registers, websites, organisations, reference lists and other sources searched or consulted to identify studies. Specify the date when each source was last searched or consulted.	P2
Search strategy	7	Present the full search strategies for all databases, registers and websites, including any filters and limits used.	P2; Table S1
Selection process	8	Specify the methods used to decide whether a study met the inclusion criteria of the review, including how many reviewers screened each record and each report retrieved, whether they worked independently, and if applicable, details of automation tools used in the process.	P3
Data collection process	9	Specify the methods used to collect data from reports, including how many reviewers collected data from each report, whether they worked independently, any processes for obtaining or confirming data from study investigators, and if applicable, details of automation tools used in the process.	P3
Data items	10a	List and define all outcomes for which data were sought. Specify whether all results that were compatible with each outcome domain in each study were sought (e.g. for all measures, time points, analyses), and if not, the methods used to decide which results to collect.	P3
	10b	List and define all other variables for which data were sought (e.g. participant and intervention characteristics, funding sources). Describe any assumptions made about any missing or unclear information.	P3
Study risk of bias assessment	11	Specify the methods used to assess risk of bias in the included studies, including details of the tool(s) used, how many reviewers assessed each study and whether they worked independently, and if applicable, details of automation tools used in the process.	P3
Effect measures	12	Specify for each outcome the effect measure(s) (e.g. risk ratio, mean difference) used in the synthesis or presentation of results.	P3
Synthesis methods	13a	Describe the processes used to decide which studies were eligible for each synthesis (e.g. tabulating the study intervention characteristics and comparing against the planned groups for each synthesis (item #5)).	P3
	13b	Describe any methods required to prepare the data for presentation or synthesis, such as handling of missing summary statistics, or data conversions.	P3
	13c	Describe any methods used to tabulate or visually display results of individual studies and syntheses.	P3
	13d	Describe any methods used to synthesize results and provide a rationale for the choice(s). If meta-analysis was performed, describe the model(s), method(s) to identify the presence and extent of statistical heterogeneity, and software package(s) used.	P3
	13e	Describe any methods used to explore possible causes of heterogeneity	P3

Section and Topic	Item #	Checklist item	Location where item is reported
		among study results (e.g. subgroup analysis, meta-regression).	
	13f	Describe any sensitivity analyses conducted to assess robustness of the synthesized results.	P3
Reporting bias assessment	14	Describe any methods used to assess risk of bias due to missing results in a synthesis (arising from reporting biases).	P3
Certainty assessment	15	Describe any methods used to assess certainty (or confidence) in the body of evidence for an outcome.	Table S2
**RESULTS**			
Study selection	16a	Describe the results of the search and selection process, from the number of records identified in the search to the number of studies included in the review, ideally using a flow diagram.	P4
	16b	Cite studies that might appear to meet the inclusion criteria, but which were excluded, and explain why they were excluded.	P4
Study characteristics	17	Cite each included study and present its characteristics.	P4-14
Risk of bias in studies	18	Present assessments of risk of bias for each included study.	P8-10
Results of individual studies	19	For all outcomes, present, for each study: (a) summary statistics for each group (where appropriate) and (b) an effect estimate and its precision (e.g. confidence/credible interval), ideally using structured tables or plots.	P12-14
Results of syntheses	20a	For each synthesis, briefly summarise the characteristics and risk of bias among contributing studies.	P12-14
	20b	Present results of all statistical syntheses conducted. If meta-analysis was done, present for each the summary estimate and its precision (e.g. confidence/credible interval) and measures of statistical heterogeneity. If comparing groups, describe the direction of the effect.	P12-14
	20c	Present results of all investigations of possible causes of heterogeneity among study results.	Figures S1–S4
	20d	Present results of all sensitivity analyses conducted to assess the robustness of the synthesized results.	Figures S1–S4
Reporting biases	21	Present assessments of risk of bias due to missing results (arising from reporting biases) for each synthesis assessed.	-
Certainty of evidence	22	Present assessments of certainty (or confidence) in the body of evidence for each outcome assessed.	Table S2
**DISCUSSION**			
Discussion	23a	Provide a general interpretation of the results in the context of other evidence.	P15,16
	23b	Discuss any limitations of the evidence included in the review.	P16,17
	23c	Discuss any limitations of the review processes used.	P16,17
	23d	Discuss implications of the results for practice, policy, and future research.	P16,17
**OTHER INFORMATION**			
Registration and protocol	24a	Provide registration information for the review, including register name and registration number, or state that the review was not registered.	P2
	24b	Indicate where the review protocol can be accessed, or state that a protocol was not prepared.	P2
	24c	Describe and explain any amendments to information provided at registration or in the protocol.	-
Support	25	Describe sources of financial or non-financial support for the review, and the role of the funders or sponsors in the review.	P18

Section and Topic	Item #	Checklist item	Location where item is reported
Competing interests	26	Declare any competing interests of review authors.	P18
Availability of data, code and other materials	27	Report which of the following are publicly available and where they can be found: template data collection forms; data extracted from included studies; data used for all analyses; analytic code; any other materials used in the review.	P18

Source: Adapted from Page et al. [39] under the CC BY 4.0 license.

Table S4. PRISMA 2020 for Abstracts Checklist

Section and Topic	Item #	Checklist item	Reported (Yes/No)
**TITLE**			
Title	1	Identify the report as a systematic review.	Yes
**BACKGROUND**			
Objectives	2	Provide an explicit statement of the main objective(s) or question(s) the review addresses.	Yes
**METHODS**			
Eligibility criteria	3	Specify the inclusion and exclusion criteria for the review.	Yes
Information sources	4	Specify the information sources (e.g. databases, registers) used to identify studies and the date when each was last searched.	Yes
Risk of bias	5	Specify the methods used to assess risk of bias in the included studies.	Yes
Synthesis of results	6	Specify the methods used to present and synthesise results.	Yes
**RESULTS**			
Included studies	7	Give the total number of included studies and participants and summarise relevant characteristics of studies.	Yes
Synthesis of results	8	Present results for main outcomes, preferably indicating the number of included studies and participants for each. If meta-analysis was done, report the summary estimate and confidence/credible interval. If comparing groups, indicate the direction of the effect (i.e. which group is favoured).	Yes
**DISCUSSION**			
Limitations of evidence	9	Provide a brief summary of the limitations of the evidence included in the review (e.g. study risk of bias, inconsistency and imprecision).	Yes
Interpretation	10	Provide a general interpretation of the results and important implications.	Yes
**OTHER**			
Funding	11	Specify the primary source of funding for the review.	Yes
Registration	12	Provide the register name and registration number.	Yes

Source: Adapted from Page et al. [39] under the CC BY 4.0 license.
